# Association Between Sleep Apnea Risk and Obesity Phenotypes in Korean Adults: A Nationwide Population-Based Study

**DOI:** 10.3390/jcm15093240

**Published:** 2026-04-24

**Authors:** Young Sang Lyu, Jun Hyung Lee, Youngmin Yoon, Jin Hwa Kim, Sang Yong Kim

**Affiliations:** 1 Department of Endocrinology and Metabolism, Chosun University Hospital, Chosun University School of Medicine, Gwangju 61453, Republic of Korea; lyu0923@chosun.ac.kr (Y.S.L.); endocrine@chosun.ac.kr (J.H.K.); 2 Department of Internal Medicine, Chosun University School of Medicine, Gwangju 61453, Republic of Korea; pp3614@chosun.ac.kr; 3 Division of Nephrology, Department of Medicine, Chosun University Hospital, Chosun University School of Medicine, Gwangju 61453, Republic of Korea; korean8503@chosun.ac.kr

**Keywords:** obstructive sleep apnea, obesity phenotypes, metabolic syndrome, STOP-Bang questionnaire, metabolic health, KNHANES, Korean adults

## Abstract

**Background/Objectives**: This study analyzes the relationship between obesity phenotypes and sleep apnea risk in the Korean population. **Methods**: This study utilized data from the Korean National Health and Nutrition Examination Survey (KNHANES) collected between 2019 and 2021 (*n* = 10,970 adults; age ≥ 40 years). Obesity phenotypes were classified into four groups based on body mass index (BMI) and the presence of metabolic syndrome: metabolically healthy normal weight (MHNW), metabolically abnormal normal weight (MANW), metabolically healthy obese (MHO), and metabolically abnormal obese (MAO). Sleep apnea risk was assessed using the STOP-Bang questionnaire, and multivariate logistic regression analyses were performed to evaluate the association between obesity phenotypes and sleep apnea. **Results**: Among the 10,970 participants, the phenotypes were as follows: MHNW, 51.1%; MANW, 10.3%; MHO, 15.8%; and MAO, 21.8%. Baseline characteristics differed significantly across phenotypes, with the metabolically unhealthy groups (MANW and MAO) being older and exhibiting more cardiometabolic risk factors than the metabolically healthy groups. The prevalence of STOP-Bang questionnaire components differed significantly across phenotypes (all *p* < 0.001), and the mean STOP-Bang score increased from MHNW to MAO. In multivariate logistic regression analyses, the odds (adjusted odds ratio [95% CI]) of high sleep apnea risk were significantly elevated in all non-MHNW phenotypes: MAO (10.27 [7.71–13.68]), MHO (6.17 [4.35–8.75]), and MANW (1.91 [1.22–2.98]). Notably, MAO conferred a significantly higher risk than MHO (OR 1.69 [1.34–2.13]), highlighting the synergy of obesity and metabolic dysfunction. Obesity phenotypes, defined by BMI and metabolic health status, were differentially associated with sleep apnea risk in Korean adults. The highest risk was observed in individuals with both obesity and metabolic syndrome, while metabolically abnormal normal-weight adults also showed a significantly increased risk. **Conclusions**: Metabolic dysfunction may contribute to sleep apnea risk beyond body size alone and may be considered in risk stratification strategies.

## 1. Introduction

Obstructive sleep apnea (OSA) is a prevalent yet frequently underrecognized sleep-related breathing disorder marked by repeated episodes of upper airway collapse during sleep, leading to intermittent hypoxia and sleep fragmentation [1]. Recent studies have suggested that OSA is closely associated with metabolic disorders and adverse health outcomes, including hypertension [2], type 2 diabetes mellitus [3], cardiovascular disease [4], and increased mortality [5], making it a significant public health concern. Despite its clinical importance, a large proportion of individuals at high risk for OSA remain undiagnosed in the general population, highlighting the need for early risk stratification [6].

Obesity is a well-established risk factor for OSA through mechanisms such as narrowing of the upper airway, increased neck circumference, and reduced lung volume [7]. However, the risk of OSA is influenced by multiple factors other than the body mass index (BMI), suggesting that obesity alone may not fully account for this heterogeneity [8]. In this context, increasing attention is being paid to body size phenotypes that integrate both adiposity and metabolic health status. Obesity phenotypes are commonly classified into metabolically healthy normal weight (MHNW), metabolically abnormal normal weight (MANW), metabolically healthy obese (MHO), and metabolically abnormal obese (MAO), and have been shown to better reflect cardiometabolic risk than BMI alone [9]. Notably, individuals with MANW may exhibit insulin resistance, visceral adiposity, and systemic inflammation, indicating that metabolic dysfunction may increase disease risk independent of body size [10,11].

However, population-based evidence regarding the association between obesity phenotypes and OSA risk remains limited, particularly in Asian populations. This is clinically relevant because Asians tend to develop metabolic abnormalities at lower BMI levels, and BMI-based definitions of obesity may underestimate the disease risk in those with MANW. Therefore, this study evaluated the relationship between obesity phenotypes, based on metabolic health and obesity status, and OSA risk.

## 2. Materials and Methods

### 2.1. Study Population

This study used data obtained from the Korean National Health and Nutrition Examination Survey (KNHANES) conducted between 2019 and 2021 by the Korea Ministry of Health and Welfare. The KNHANES is an ongoing, nationally representative survey designed to evaluate the health and nutritional status of the Korean population using a stratified, multistage probability sampling design to ensure representativeness across regions, sexes, and age groups [12]. The survey includes standardized health interviews, nutritional surveys, and physical examinations conducted by trained healthcare professionals.

**Figure 1:**
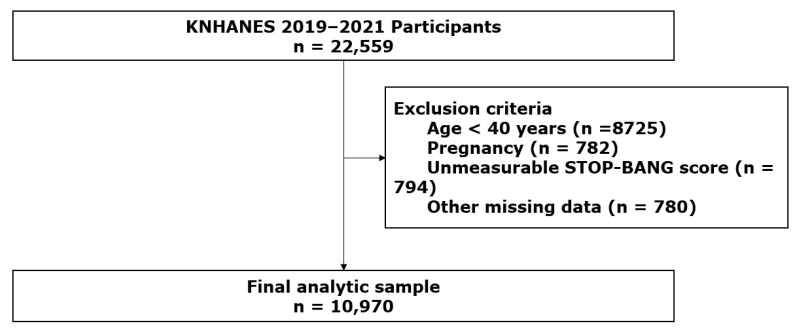
Flow diagram of the study population selected from KNHANES 2019–2021.

A total of 22,948 individuals participated in KNHANES from 2019 to 2021. Among them, adults aged ≥ 40 years with available data on anthropometric measurements, metabolic parameters, and sleep apnea-related questionnaire items were considered eligible for the present analysis. Participants with missing key variables required to define obesity phenotypes or assess sleep apnea risk were excluded. After applying the exclusion criteria, 10,970 participants were included in the final sample (Figure 1).

Ethical approval for this study was obtained from the Institutional Review Board of Chosun University Hospital (approval no. 2026-03-001; 4 March 2026). All participants provided written informed consent, and the study adhered to the principles of the Declaration of Helsinki.

### 2.2. Measurements and Variable Definitions

#### 2.2.1. Anthropometric and Laboratory Assessments

Anthropometric data were collected by trained personnel using standardized measurement protocols. Body weight and height were measured with calibrated devices, and BMI was calculated as body weight (kg) divided by height squared (m^2^). Waist circumference was assessed at the midpoint between the lower rib margin and the iliac crest.

Blood pressure was assessed after a minimum of 5 min of seated rest using a standard mercury sphygmomanometer, and the mean of two results was used for analysis. Fasting blood samples were obtained following at least 8 h of overnight fasting. Levels of fasting plasma glucose, triglycerides, and high-density lipoprotein cholesterol (HDL-C) were measured using an automated biochemical analyzer (Hitachi Automatic Analyzer 7600; Hitachi, Tokyo, Japan). Glycated hemoglobin (HbA1c) was measured using high-performance liquid chromatography (Tosoh G8; Tosoh Bioscience, Tokyo, Japan).

Information on sociodemographic characteristics and lifestyle factors, including residential area, household income, education level, smoking status, alcohol intake, total energy intake and physical activity were obtained through structured self-administered questionnaires.

#### 2.2.2. Definition of Obesity Phenotypes

Metabolic syndrome was defined based on the presence of three or more of the following criteria: (1) central obesity, determined by a waist circumference ≥90 cm in men or ≥85 cm in women according to the Korean Society for the Study of Obesity criteria [13]; (2) hypertriglyceridemia (triglyceride level ≥ 150 mg/dL) or current treatment for elevated triglyceride level; (3) reduced HDL-C levels (<40 mg/dL in men or <50 mg/dL in women) or use of lipid-lowering medications; (4) elevated blood pressure (systolic ≥ 130 mmHg and/or diastolic ≥ 85 mmHg) or current use of antihypertensive drugs; (5) impaired fasting glucose (≥100 mg/dL) or treatment with glucose-lowering medications.

Participants were subsequently categorized into four body size phenotypes based on BMI according to Asian-specific criteria [14] and metabolic syndrome status: MHNW, defined as BMI of 18.5–24.9 kg/m^2^ without metabolic syndrome; MANW, normal BMI with metabolic syndrome; MHO, BMI ≥ 25 kg/m^2^ without metabolic syndrome; and MAO, BMI ≥ 25 kg/m^2^ with metabolic syndrome.

#### 2.2.3. Assessment of Sleep Apnea Risk

The risk of OSA was evaluated using the STOP-Bang questionnaire, a validated screening instrument widely applied in epidemiological and clinical research [15]. The STOP-Bang score comprises eight components: snoring, daytime fatigue, observed apnea, hypertension, BMI, age, neck circumference, and sex. Based on the established scoring thresholds, participants were categorized into low-, intermediate-, and high-risk groups for sleep apnea. For the primary analyses, individuals in the high-risk group were compared with those in the low-to-intermediate-risk group.

### 2.3. Statistical Analysis

All statistical analyses accounted for the complex sampling design, stratification, and sample weights of the KNHANES dataset in accordance with the analytic guidelines provided by the Korea Disease Control and Prevention Agency. Continuous variables are reported as means ± standard error, whereas categorical variables are presented as weighted proportions. Baseline characteristics were compared according to the obesity phenotype groups and sleep apnea risk categories using complex sample general linear models for continuous variables and Rao–Scott chi-square tests for categorical variables. Multivariable logistic regression models were applied to evaluate the association between obesity phenotypes and a high risk of sleep apnea. The models were adjusted for potential confounders, including age, sex, sociodemographic factors (residential area, household income, and educational level), and lifestyle behaviors (smoking status, alcohol consumption, regular physical activity, and total energy intake). Adjusted odds ratios (ORs) and 95% confidence intervals (CIs) were estimated using the MHNW group as the reference category. All statistical analyses were performed using SPSS (version 25.0; IBM Corp., Armonk, NY, USA), and a two-sided *p*-value < 0.05 was considered statistically significant.

## 3. Results

### 3.1. Baseline Characteristics According to Obesity Phenotypes

The final analysis included 10,970 participants categorized into four body size phenotype groups: MHNW (*n* = 5616), MANW (*n* = 1225), MHO (*n* = 1731), and MAO (*n* = 2398) (Table 1). Significant differences in demographic, metabolic, and sleep-related characteristics were observed across the phenotype groups (all *p* < 0.001). Participants in the MANW group were the oldest, whereas those in the MHNW and MHO groups were relatively younger. The proportion of males was highest in the MAO group and lowest in the MHNW group. As expected, BMI and waist circumference increased progressively across the phenotype spectrum, with the highest values observed in the MAO group. The metabolic profiles differed markedly among the groups. The MANW and MAO groups had higher fasting plasma glucose and HbA1c levels than the MHNW and MHO groups. Triglyceride levels were substantially higher and HDL-C levels were lower in the metabolically abnormal group, indicating a more adverse cardiometabolic profile.

The prevalence of individual STOP-Bang questionnaire components differed significantly across the body size phenotypes (all *p* < 0.001) (Table 2). Snoring, observed apnea, and neck circumference > 40 cm progressively increased from MHNW to MAO, with the highest prevalence observed in the MAO group. Tiredness was also more prevalent in the metabolically unhealthy groups, particularly MAO, although the difference across groups was less pronounced than that in the other components. Hypertension showed a marked increase according to metabolic status and was substantially higher in the MANW and MAO groups than in the MHNW group. As expected, the proportion of participants with BMI > 30 kg/m^2^ was limited to obese phenotypes, with a higher prevalence in MAO than in MHO. Older age (≥50 years) was the most prevalent factor in the MANW group, suggesting a potential contribution of age to metabolic dysfunction in normal-weight individuals. Male sex was also more common in the metabolically unhealthy and obese phenotypes, with the highest proportion in the MAO group. Overall, the mean STOP-Bang score increased stepwise across phenotypes, from 1.8 in MHNW to 3.0 in MAO, indicating a progressively higher risk of sleep apnea with worsening metabolic health and obesity status.

### 3.2. Characteristics According to Sleep Apnea Risk Category

When participants were stratified by sleep apnea risk, 6769 (61.7%) were classified as low risk, 3532 (32.2%) as intermediate risk, and 669 (6.1%) as high risk (Table 3). Individuals in the high-risk group were predominantly male and had a significantly higher BMI and waist circumference than those in the low- and intermediate-risk groups (*p* < 0.001). Cardiometabolic parameters worsened with increasing sleep apnea risk. Participants in the high-risk group showed higher fasting glucose, HbA1c, triglyceride, and blood pressure levels, and lower HDL-C levels, indicating a more adverse metabolic profile (all *p* < 0.001). The distribution of obesity phenotypes differed significantly across sleep apnea risk categories (*p* < 0.001). The proportion of patients with the MAO phenotype markedly increased with increasing sleep apnea risk, whereas the prevalence of the MHNW phenotype substantially decreased. The MHO phenotype showed a modest increase across the risk groups, whereas the MANW phenotype demonstrated a relatively smaller variation.

### 3.3. Association Between Obesity Phenotypes and High Risk of Sleep Apnea

Multivariate logistic regression analyses were performed to evaluate the association between obesity phenotypes and a high risk of sleep apnea after adjusting for age, sex, sociodemographic factors, and lifestyle behaviors (Table 4). Compared with the MHNW group, the odds of being at high risk for sleep apnea were significantly higher in the MAO (adjusted OR 10.27, 95% CI 7.71–13.68), MHO (adjusted OR 6.17, 95% CI 4.35–8.75), and MANW (adjusted OR 1.91, 95% CI 1.22–2.98) groups. Additional comparisons between the phenotypes revealed that the MAO group had a significantly higher risk than the MHO group (OR 1.69, 95% CI 1.34–2.13). Furthermore, the MAO group had a higher risk than the MANW group (OR 5.15, 95% CI 3.56–7.45), and the MHO group had a higher risk than the MANW group (OR 3.33, 95% CI 2.22–5.01).

## 4. Discussion

In this nationally representative study of Korean adults aged ≥ 40 years, both obesity and metabolic dysfunction were associated with an increased risk of sleep apnea. The highest risk was observed in the MAO group, whereas even those in the MANW group showed a significantly higher risk than those in the MHNW group. These findings suggest that in Korean adults, metabolic dysfunction, in addition to obesity, may play an important role in determining the risk of sleep apnea and highlight the importance of obesity phenotype-based approaches.

Obesity is a well-established risk factor for the development and progression of OSA [1,7]. Consistent with this concept, the MHO phenotype in our study also showed a significantly higher risk of sleep apnea compared with the MHNW group, suggesting that excess adiposity itself may substantially contribute to sleep-disordered breathing, even in the absence of overt metabolic abnormalities. Recent large-scale epidemiological evidence supports this association. A meta-analysis showed that being overweight and obese was significantly associated with OSA, with a higher BMI in adults with OSA and a higher apnea–hypopnea index (AHI) in obese individuals than in non-obese individuals in both adults and children [16]. Another population-based prospective study demonstrated that a 10% weight gain was associated with an approximately 32% increase in AHI and sixfold higher odds of developing moderate-to-severe sleep-disordered breathing, whereas a 10% weight loss was associated with a 26% reduction in AHI [17]. Together, these findings suggest that excess adiposity is not only associated with OSA cross-sectionally but may also contribute to its progression over time.

One of the most clinically important findings of this study was the significantly increased risk of sleep apnea in the MANW group. Individuals with normal body weight are generally considered less likely to have OSA and are therefore less likely to be targeted for screening in routine clinical practice [10]. However, our findings suggest that metabolic syndrome itself may identify a subgroup of normal-weight adults at an increased risk of sleep apnea. This implies that metabolic abnormalities, such as insulin resistance, dyslipidemia, hyperglycemia, and elevated blood pressure, may contribute to OSA risk independent of obesity [18]. Similarly, previous studies have demonstrated a close link between OSA and metabolic dysfunction, including metabolic syndrome and insulin resistance, even after accounting for excess body weight [18,19,20]. In particular, metabolically unhealthy normal-weight individuals may harbor increased visceral adiposity and systemic inflammatory burden despite having a non-obese BMI, which may adversely affect upper airway mechanics and ventilatory control [21,22]. Therefore, our results support the view that assessments based solely on BMI may underestimate OSA risk and that metabolic syndrome should also be incorporated into risk stratification and screening strategies, even in normal-weight adults.

The MAO phenotype showed the highest odds of high sleep apnea risk, exceeding those observed in both the MHO and MANW groups. This suggests that the coexistence of obesity and metabolic syndrome may exert additive or even synergistic effects on sleep apnea risk. Notably, the higher risk observed in the MAO group than in the MHO group highlights the amplifying role of metabolic abnormalities beyond excess adiposity alone. At the same time, the greater risk in the MAO group than in the MANW group indicates that body size and mechanical factors remain important contributors to sleep-disordered breathing. These findings are consistent with previous epidemiological evidence demonstrating an association between metabolic syndrome and OSA. A nationwide population-based study of Korean adults reported that metabolic syndrome and its individual components were significantly associated with an increased likelihood of OSA [8]. However, that study primarily evaluated metabolic syndrome alone and did not consider combined obesity–metabolic phenotypes. In contrast, our study jointly evaluated obesity and metabolic health status, allowing a more comprehensive assessment of how excess adiposity and metabolic dysfunction interact to influence sleep apnea risk. Taken together, these findings suggest that obesity and metabolic dysfunction jointly contribute to the development of sleep apnea and that individuals with both conditions represent a particularly high-risk population.

Several mechanisms may explain the observed association between obesity phenotypes and sleep apnea risk. First, excess adiposity, particularly central and visceral fat accumulation, may mechanically promote upper airway narrowing and reduce lung volume, thereby increasing airway collapsibility during sleep [7]. Fat deposition around the neck and abdomen can also increase the mechanical loading of the respiratory system and reduce functional residual capacity, further predisposing individuals to obstructive events [23]. Second, metabolic syndrome is closely linked to insulin resistance, dysglycemia, dyslipidemia, and low-grade systemic inflammation, all of which may adversely affect ventilatory control and upper airway neuromuscular function [24]. These metabolic abnormalities may also contribute to endothelial dysfunction and altered autonomic regulation, potentially increasing susceptibility to sleep-disordered breathing [25]. Third, intermittent hypoxia and sleep fragmentation associated with OSA may further aggravate metabolic dysfunction through the activation of sympathetic pathways, oxidative stress, and inflammatory responses, suggesting a bidirectional relationship between OSA and adverse metabolic health [26]. Taken together, these mechanisms provide a plausible biological explanation for the higher sleep apnea risk observed across metabolically unhealthy phenotypes.

These findings have significant implications for public health. As sleep apnea remains under-recognized and undertreated in the general population, risk stratification strategies that extend beyond BMI alone may help improve the early identification of individuals at an elevated risk. In particular, incorporating indicators of metabolic health with anthropometric measures may facilitate the identification of individuals who might otherwise not be considered for further evaluation. This approach may be particularly relevant in Korean and other Asian populations, where metabolic abnormalities can occur, even among individuals with a relatively low BMI.

This study had several strengths. First, the analysis was based on a large, nationally representative sample of Korean adults derived from a nationwide survey, which enhances the generalizability of the findings. Second, by classifying participants according to combined obesity–metabolic phenotypes, our study provided a more comprehensive assessment of sleep apnea risk than analyses based solely on BMI. Third, we adjusted for a wide range of sociodemographic and lifestyle-related variables, including major behavioral factors, which helped reduce the likelihood that the observed associations were explained by major confounders.

This study has several limitations that warrant consideration when interpreting the findings. First, owing to the cross-sectional design of the study, causal relationships could not be established. Although metabolic dysfunction may contribute to an increased risk of sleep apnea, sleep apnea itself may worsen metabolic abnormalities through intermittent hypoxia, sympathetic activation, and sleep fragmentation. Second, sleep apnea risk was assessed using the STOP-Bang questionnaire rather than polysomnography. Therefore, our analysis reflects the risk of sleep apnea rather than clinically confirmed OSA. Although the STOP-Bang is a widely used and validated screening tool, the possibility of misclassification cannot be excluded [27]. Third, the classification of obesity phenotypes was primarily based on BMI and metabolic components and did not include direct measures of body fat distribution, insulin resistance, or inflammatory biomarkers, which may provide additional insight into underlying mechanisms. Fourth, because the STOP-Bang questionnaire incorporates BMI, age, sex, and hypertension, which are also related to the definition of obesity phenotypes and covariates, the observed associations may have been partially influenced by shared components. Fifth, serum albumin and total protein, which reflect nutritional status, were not available in the KNHANES dataset, limiting the analysis. Therefore, residual confounding related to nutritional status may have been present. Finally, despite adjusting for multiple covariates, residual confounding by unmeasured or incompletely measured factors cannot be entirely ruled out.

## 5. Conclusions

Obesity phenotypes, defined by both BMI and metabolic health status, were differentially associated with sleep apnea risk. The highest risk was observed in individuals with both obesity and metabolic syndrome, whereas metabolically unhealthy, normal-weight adults showed a significantly increased risk. These findings indicate that metabolic dysfunction may play an important role in sleep apnea risk beyond body size alone. Incorporating metabolic health indicators into screening strategies may improve the early identification of individuals at high risk of sleep apnea, particularly in Korean populations, where metabolic abnormalities often occur at lower BMI levels.

## Figures and Tables

**Table 1:** Characteristics of the study population according to body size phenotypes.

	MHNW	MANW	MHO	MAO	*p*-Value
Number (%)	5616 (51.1)	1225 (10.3)	1731 (15.8)	2398 (21.8)	
Sex (male, %)	38.2	46.3	47.2	50.2	<0.001
Age (years)	58.29 ± 11.61	64.94 ± 10.72	58.89 ± 11.42	60.28 ± 11.37	<0.001
BMI (kg/m^2^)	21.89 ± 1.90	23.13 ± 1.50	27.28 ± 2.23	28.01 ± 2.54	<0.001
WC (cm)	78.67 ± 6.77	85.73 ± 6.16	92.30 ± 7.29	95.62 ± 6.73	<0.001
SBP (mmHg)	117.74 ± 16.24	132.29 ± 16.47	120.24 ± 13.09	129.76 ± 15.03	<0.001
DBP (mmHg)	73.78 ± 9.13	77.79 ± 10.26	75.80 ± 8.63	79.88 ± 10.21	<0.001
MAP (mmHg)	88.43 ± 8.51	95.96 ± 9.92	90.61 ± 7.54	96.51 ± 9.23	<0.001
FPG (mg/dL)	98.22 ± 17.69	116.08 ± 28.02	101.25 ± 18.60	116.12 ± 29.09	<0.001
FPG (mmol/L)	5.46 ± 0.98	6.45 ± 1.56	5.63 ± 1.03	6.45 ± 1.62	<0.001
HbA1c (%)	5.74 ± 0.65	6.33 ± 1.07	5.85 ± 0.69	6.32 ± 1.05	<0.001
LDL-C (mg/dL)	117.13 ± 38.07	108.74 ± 37.40	126.08 ± 31.48	113.02 ± 36.40	<0.001
HDL-C (mg/dL)	56.03 ± 12.75	43.40 ± 9.94	53.08 ± 10.44	44.43 ± 9.91	<0.001
TG (mg/dL)	103.52 ± 62.27	200.16 ± 163.31	108.11 ± 57.62	190.53 ± 134.42	<0.001
Creatinine (mg/dL)	0.79 ± 0.29	0.84 ± 0.32	0.82 ± 0.19	0.84 ± 0.24	<0.001
Family income percentile (%) ^a^					
<25	19.0	29.9	18.5	24.4	
25–50	23.9	28.9	27.3	24.7	
50–75	26.5	20.7	27.5	25.1	
≥75	30.6	20.4	26.7	25.9	<0.001
More than high school education (%)	70.2	47.8	64.4	57.7	<0.001
Residence in urban area (%)	79.7	70.5	78.2	74.4	<0.001
Smoking					
Never	63.7	56.2	58.3	53.9	
Past	22.2	25.4	28.2	29.7	
Current	14.1	18.2	13.3	16.4	<0.001
Alcohol drinking (Yes, %)	6.2	9.3	8.0	11.2	<0.001
Regular exercise ^a^ (yes, %)	40.4	34.1	42.0	34.9	<0.001

Data are expressed as the mean ± SE for continuous variables and as weighted percentages for categorical variables. BMI, body mass index; DBP, diastolic blood pressure; FPG, fasting plasma glucose; MANW, metabolic abnormal normal weight; MAO, metabolic abnormal obese; MHNW, metabolic healthy normal weight; MHO, metabolic healthy obese; SBP, systolic blood pressure; TG, triglyceride; WC, waist circumference. ^a^ Regular exercise was defined as engaging in physical activity for at least 2.5 h of moderate-intensity physical activity per week or 1.25 h of high-intensity physical activity.

**Table 2:** Prevalence of STOP-Bang questionnaire components according to body size phenotypes.

	MHNW	MANW	MHO	MAO	*p*-Value
Snoring	12.7	18.1	24.8	29.7	<0.001
Tiredness	28.8	31.3	31.3	32.8	<0.001
Observed apnea	6.3	7.1	11.4	13.8	<0.001
hypertension	22.0	46.7	37.8	50.9	<0.001
BMI > 30 kg/m^2^	0	0	10.7	18.1	<0.001
Age > 50 years	71.8	90.5	73.4	78.1	<0.001
Neck circumference > 40 cm	0.5	1.9	15.1	23.5	<0.001
Male sex	38.2	46.3	47.2	50.2	<0.001
STOP-BANG score	1.8	2.4	2.5	3.0	<0.001

Data are expressed as weighted percentages for categorical variables.

**Table 3:** Characteristics of the study population according to sleep apnea risk category.

	Low Risk	Intermediate Risk	High Risk	*p*-Value
Number (%)	6769	3532	669	
Sex (male, %)	25.9	67.2	90.6	<0.001
Age (years)	57.42 ± 11.69	63.58 ± 10.65	60.00 ± 9.94	<0.001
BMI (kg/m^2^)	23.39 ± 3.02	25.08 ± 3.44	27.98 ± 3.83	<0.001
WC (cm)	82.04 ± 8.81	89.29 ± 8.95	97.39 ± 9.39	<0.001
SBP (mmHg)	119.84 ± 16.89	126.52 ± 15.58	126.36 ± 13.98	<0.001
DBP (mmHg)	75.08 ± 9.54	76.68 ± 9.76	79.84 ± 10.66	<0.001
FPG (mg/dL)	101.24 ± 20.84	109.05 ± 25.77	115.21 ± 29.18	<0.001
HbA1c (%)	5.83 ± 0.77	6.11 ± 0.94	6.32 ± 1.05	<0.001
LDL-C (mg/dL)	117.28 ± 36.69	112.06 ± 37.22	101.34 ± 34.63	<0.001
HDL-C (mg/dL)	53.79 ± 12.89	48.69 ± 11.87	45.09 ± 10.00	<0.001
TG (mg/dL)	123.23 ± 94.22	147.05 ± 119.03	175.01 ± 123.98	<0.001
Creatinine (mg/dL)	0.75 ± 0.20	0.89 ± 0.36	0.93 ± 0.23	<0.001
Family income percentile (%) ^a^				
<25	18.8	26.5	19.3	
25–50	24.5	26.5	25.3	
50–75	27.1	23.2	25.7	
≥75	29.6	23.9	29.7	<0.001
More than high school education (%)	67.9	55.6	70.1	<0.001
Residence in urban area (%)	79.5	73.6	73.5	<0.001
Smoking				
Never	72.7	42.8	20.8	<0.001
Past	16.0	37.4	52.2	<0.001
Current	11.2	19.7	26.9	<0.001
Alcohol drinking (Yes, %)	5.2	11.0	19.4	<0.001
Regular exercise ^a^ (yes, %)	39.2	37.7	39.2	0.345
Metabolic phenotype				
MHNW	61.5	38.6	13.5	
MANW	10.3	13.4	7.6	
MHO	13.6	18.4	23.9	
MAO	14.6	29.6	55.0	<0.001

Data are expressed as the mean ± SE for continuous variables and as weighted percentages for categorical variables. BMI, body mass index; DBP, diastolic blood pressure; eGFR, estimated glomerular filtration rate; FPG, fasting plasma glucose; MANW, metabolic abnormal normal weight; MAO, metabolic abnormal obese; MHNW, metabolic healthy normal weight; MHO, metabolic healthy obese; SBP, systolic blood pressure; TG, triglyceride; WC, waist circumference. ^a^ Regular exercise was defined as engaging in physical activity for at least 2.5 h of moderate-intensity physical activity per week or 1.25 h of high-intensity physical activity.

**Table 4:** Association between sleep apnea risk and body size phenotypes.

	Fully Adjusted OR (95% CI)				
	MAO		MANW		MHO
MAO/MHNW		MANW/MHNW		MHO/MHNW	
Low/intermediate risk	Reference	Low/intermediate risk	Reference	Low/intermediate risk	Reference
High risk	10.274 (7.714–13.684)	High risk	1.906 (1.217–2.983)	High risk	6.168 (4.346–8.753)
MAO/MANW		MHO/MANW			
Low/intermediate risk	Reference	Low/intermediate risk	Reference		
High risk	5.151 (3.562–7.449)	High risk	3.333 (2.216–5.013)		
MAO/MHO					
Low/intermediate risk	Reference				
High risk	1.689 (1.337–2.132)				

CI, confidence interval; MANW, metabolic abnormal normal weight; MAO, metabolic abnormal obese; MHNW, metabolic healthy normal weight; MHO, metabolic healthy obese; OR, odds ratio. Logistic models are adjusted for age, sex, baseline metabolic profile (systolic blood pressure, diastolic blood pressure, HbA1c, LDL-Cholesterol, triglyceride, creatinine), sociodemographic factors (place of residence, family income, and education), lifestyle behaviors (smoking status, alcohol consumption, regular exercise).

## Data Availability

Information can be accessed from the Korea National Health and Nutrition Examination Survey (KNHANES), which is organized by the Korea Centers for Disease Control and Prevention (KCDCP). The data is freely available on the KCDCP website (https://www.kdca.go.kr/eng/4428/subview.do), accessed on 21 February 2026.

## Abbreviations

AHI	apnea–hypopnea index
BMI	body mass index
HDL-C	high-density lipoprotein-cholesterol
KNHANES	Korean National Health and Nutrition Examination Survey
MANW	metabolically abnormal normal weight
MAO	metabolically abnormal obese
MHNW	metabolically healthy normal weight
MHO	metabolically healthy obese
OSA	obstructive sleep apnea
